# Variation of Shrinkage Strain within the Depth of Concrete Beams

**DOI:** 10.3390/ma8115421

**Published:** 2015-11-16

**Authors:** Jong-Hyun Jeong, Yeong-Seong Park, Yong-Hak Lee

**Affiliations:** Department of Civil Engineering, Konkuk University, 120 Neungdong-ro Gwangjin-gu, Seoul 143-701, Korea; kolapot@konkuk.ac.kr (J.-H.J.); parkys77@konkuk.ac.kr (Y.-S.P.)

**Keywords:** concrete, aggregate concentration, shrinkage, time-dependent beam test

## Abstract

The variation of shrinkage strain within beam depth was examined through four series of time-dependent laboratory experiments on unreinforced concrete beam specimens. Two types of beam specimens, horizontally cast and vertically cast, were tested; shrinkage variation was observed in the horizontally cast specimens. This indicated that the shrinkage variation within the beam depth was due to water bleeding and tamping during the placement of the fresh concrete. Shrinkage strains were measured within the beam depth by two types of strain gages, surface-attached and embedded. The shrinkage strain distribution within the beam depth showed a consistent tendency for the two types of gages. The test beams were cut into four sections after completion of the test, and the cutting planes were divided into four equal sub-areas to measure the aggregate concentration for each sub-area of the cutting plane. The aggregate concentration increased towards the bottom of the beam. The shrinkage strain distribution was estimated by Hobbs’ equation, which accounts for the change of aggregate volume concentration.

## 1. Introduction

In the time-dependent analysis of reinforced concrete flexural members, the shrinkage strain curve is commonly obtained by multiplying the standard form of the shrinkage function by correction factors to account for non-standard conditions during the curing process, mixture proportions, ambient temperature, and relative humidity [1,2]. Once the shrinkage strain curve is determined, the distribution of shrinkage strain within the beam depth is assumed to be uniform [3,4,5,6,7]. However, time-dependent shrinkage tests on concrete beams show that the magnitude of the shrinkage strain varies within the beam depth. It is largest in the top region of the test beam, and lowest in the bottom region. The variation of shrinkage strain within the beam depth is explained by two effects: water bleeding and aggregate sedimentation during placing work for the fresh concrete [8,9,10,11,12], and the diffusion-based drying proceeding from the surface into the interior of the concrete [13,14,15,16,17,18]. In this paper, the variation of shrinkage strain within the beam depth is examined with regard to the first effect: phenomena occurring during the concrete casting work.

Shrinkage in concrete normally depends on three factors: the water-cement ratio, the aggregate volume concentration, and the aggregate stiffness [1,2,9,10,18]. The change in water-cement ratio during the work to place concrete occurs due to the water bleeding, which decreases the concrete strength in the upper region of the beam specimen and lowers the aggregates [12]. Water bleeding is more or less interrelated with the change in aggregate volume concentration if it is taken into consideration that the amount of water bled is approximately equal to the aggregate sediment in the fresh concrete. A change in aggregate volume concentration is also produced when the fresh concrete is tamped. It is normally understood that the changes in the aggregate volume concentration and the aggregate stiffness cause the change in shrinkage, because shrinkage in concrete is restrained by the aggregate particles when the paste fraction shrinks depending on the aggregate stiffness [1,9,10]. Through this process, non-uniform values of the water-cement ratio (w/c) and aggregate volume concentration within the depth of the concrete beam result in the variation of shrinkage strain within the beam depth. In this paper, the distribution of shrinkage strain within the beam depth was investigated from a macroscopic point of view with regard to the aggregate volume concentration and the bleeding phenomenon, rather than from a microscopic point of view, considering the reaction process of the concrete constituents.

Time-dependent shrinkage tests on concrete beams were carried out with 1 m long unreinforced concrete beam specimens and 2 m long unreinforced double-T type concrete beams. The 1 m long beam specimens were cast using two types of formworks, one placed horizontally, the other vertically, to examine if the shrinkage variation within the beam depth is due to the water bleeding and tamping during placement of the fresh concrete. Variation of shrinkage strain within the beam depth was observed in the horizontally cast beam specimens, increasing toward the top of the beam. This indicates that the shrinkage variation within the beam depth is caused by water bleeding and tamping when the concrete is fresh. The amount of water bleeding is interrelated with the change of aggregate volume concentration according to [8,9,10,11,12,18]. The test beam specimens were cut through the beam depth into three parts, and the aggregate areas were measured in four equal sub-areas of each cutting plane to examine the variation of aggregate concentration within the beam depth. Though the process for estimating the aggregate concentration variation was rough and time-consuming, it was worthwhile for observing the increase of aggregate concentration toward the bottom of the beam depth. Another aspect in connection with the shrinkage variation is the change of w/c ratio within the beam depth due to the water bleeding; the ratio becomes non-uniform, increasing toward the top of the beam. The effect of the non-uniform state of the w/c ratio on the shrinkage variation within the beam depth is accounted for, to some extent, in terms of the aggregate concentration because the upward water bleeding causes sedimentation of aggregates. Tamping during placement of the fresh concrete is also a factor that influences the shrinkage variation within the beam depth according to [11,12]. Experimental observations of [9,10,11,12] indicate that tamping the fresh concrete during casting work increases the water bleeding, which is interrelated with the sedimentation of aggregates. The shrinkage variation observed within the beam depth in this paper can be attributed to three causes: water bleeding, non-uniform w/c ratio, and aggregate sedimentation. This paper mainly focuses on identifying the shrinkage variation, and characterizing the interrelation between the variations of shrinkage and aggregate concentration within the beam depth.

The data was interpreted with the shrinkage prediction equation of Hobbs [10,11] to characterize the relationship between the variations of shrinkage and aggregate concentration. The measured shrinkage strains within the beam depth were predicted by Hobbs [10] and ACI 209.2R-08 [1] based on the mix proportions and the measured aggregate concentrations. The measured shrinkage strain was decomposed into uniform and skewed parts with reference to the centroid of the test beam specimen to evaluate the degree of shrinkage variation within the beam depth. For this purpose, regression analyses were performed on the uniform and skewed parts of shrinkage strains to obtain the shrinkage parameters for the ACI code-specified shrinkage prediction formula. A comparison of the shrinkage parameters indicated that the skewed part of the shrinkage strain was sufficiently large to be considered in the time-dependent analysis and experiments on concrete structures.

## 2. Experimental Setup

### 2.1. Experimental Plan

**Figure 1:**
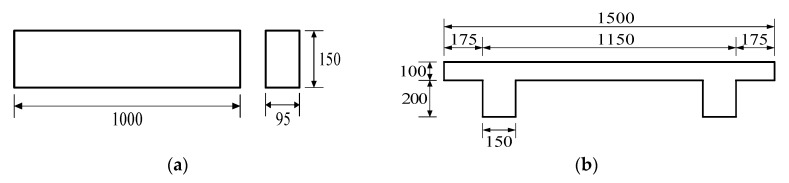
Two types of test beam specimens: (**a**) Dimension of rectangular concrete beam (**b**) Cross-sectional dimension of double-T beam (dimensions in mm).

Four sets of time-dependent laboratory experiments, A, B, C, and D were sequentially conducted on unreinforced concrete beam specimens. The distribution of shrinkage strain was measured within the depth of the test beam specimens. Three types of specimens were used for the four sets of time-dependent shrinkage experiments: rectangular concrete beams (1 m long × 150 mm high × 95 mm wide) for test sets A, B, and C; a double-T concrete beam (2 m long × 300 mm high × 1.5 m wide) for test set D; and cylindrical specimens (150 mm diameter and 150 mm height) for the compressive strengths and elastic moduli of concretes, and the shrinkage strain for the four test sets. Four rectangular beam specimens were tested for each of test series A and B. For test series C, six rectangular beam specimens were tested, where three beam specimens were cast using vertically placed formworks and the other three specimens were cast using horizontally placed formworks. A time-dependent shrinkage experiment on the double-T beam of test set D was not anticipated during the planning stage of the experiment program. The double-T beam shrinkage test was a part of a time-dependent experiment on a 9 m long two-span prestressed curved double-T beam that aimed to investigate the time-dependent torsional behavior of a prestressed curved flexural member under torsion. The 2 m long double-T beam was tested in the experimental program to determine the time-dependent development of shrinkage strain within the beam depth of the two-span double-T beam. The test results are presented in the context of the other three test sets to provide more experimental evidence for the shrinkage variation, including different types of beam structures. Figure 1a,b shows the dimensions of two types of test beam specimens.

Concrete for the test series A, B, and C were designed to have a compressive strength 28 MPa at the age of 28 days and a slump value of 180 mm, and were cast in the laboratory. The fresh concrete was poured into the formwork and tamped by a vibrating tamper at three locations (at the two ends and at mid-span of the formwork), tamping each location for 4–5 s. The void created in the formwork after tamping was refilled with fresh concrete, and tamped in the same way as the first tamping. In the case of test set D, ready-mixed concrete was used and the mix design included the maximum aggregate size and slump value as provided by the concrete manufacturer. Tamping the fresh concrete in this case was performed in the same way as in test series A, B, and C except that the tamping period was 7–8 s. The concrete mix proportions for the four sets of experiments are listed in Table 1.

**Table 1:** Concrete mix proportions for the four sets of experiments.

Test Sets	Max Aggr. Size (mm)	W/C Ratio (%)	Slump (mm)	Unit Weight, (N/m^3^)			
				Water	Cement	Fine Agg.	Coarse Aggr.
A	20	57	200	2116	3720	9447	7608
B	20	54	180	2072	3837	9615	7647
C	20	55	150	2005	3714	9771	7771
D	25	42	150	1559	3824	9591	6992

The test beam specimens for series A and C were cut through in three locations into four parts after tests, and the cutting plane was divided into four equal areas with identical heights. Aggregate concentrations were measured in each sub-area of the cutting plane. To measure the aggregate areas, photos were taken of the cutting planes and stored in a computer. The photos were divided into a number of pixels, and the aggregate areas and locations were measured by counting the number of pixels included in aggregates. The location of the aggregates was used to calculate the centroid of the aggregates after sedimentation for each cutting plane.

### 2.2. Measuring Gages

**Figure 2:**
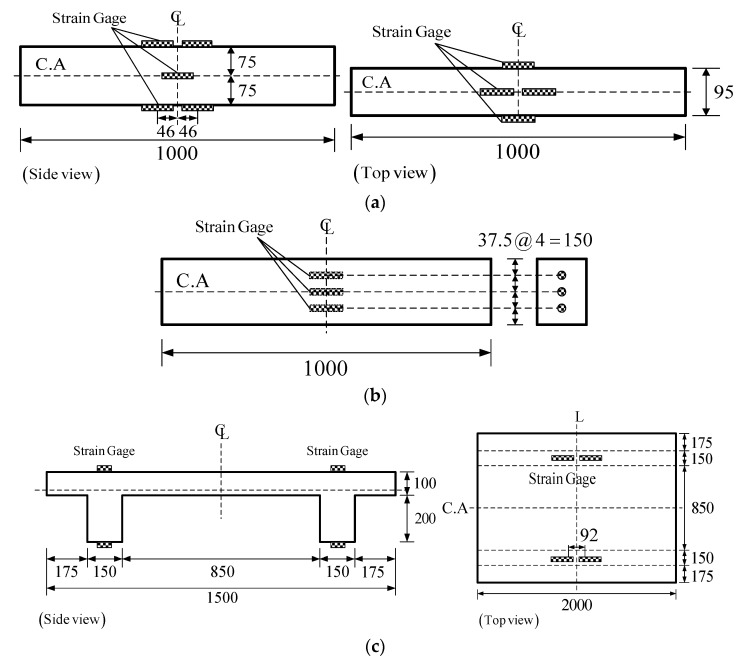
Locations of surface-attached and embedded strain gages: (**a**) Surface-attached strain gages (Rectangular beam) (**b**) Embedded strain gages (dimensions in mm) (**c**) Surface-attached strain gages.

Surface-attached strain gages were used to measure the shrinkage strains for the horizontally cast beam specimens of the test series A, B, C, and D. For test series A, B, and C, the gages were attached at the three locations within the beam depth shown in Figure 2a: top surface, mid-height surface, and bottom surface. For series C, three beam specimens used surface-attached gages, while the other three used embedded gages as well as surface-attached gages. The type and characteristics of surface-attached and embedded strain gages are listed in Table 2.

**Table 2:** Types and characteristics of surface-attached and embedded strain gages.

Type	Model (Tokyosokki)	Gage Length	Gage Width	Backing Width	Backing Thickness	Resistance (Ω)
Surface-attached	PL-10-11	60	1	8	–	120
Embedded	PMFL-60-2LT	60	1	ϕ 8	ϕ 4	120

**Figure 3:**
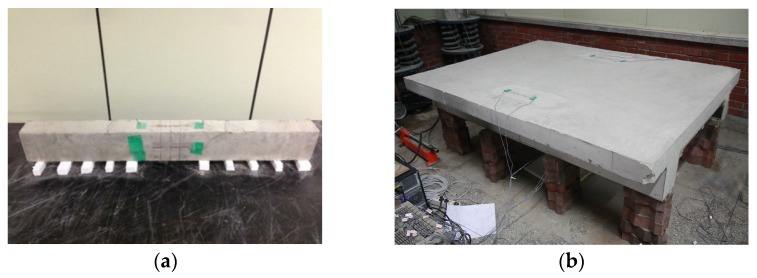
Specimen setups: (**a**) Rectangular beam (**b**) Double-T beam.

The embedded gages were installed before concrete casting at three locations inside the formworks (Figure 2b): 37.5 mm below the top surface, mid-height, and 37.5 mm above the bottom surface. The embedded gages were fixed at their locations with wire so as to maintain their positions during pouring and tamping of the fresh concrete. For test series D, surface-attached gages were located at the top surface of the flange and the bottom surfaces of the left and right webs as shown in Figure 2c. Figure 3a,b show the rectangular and double-T beam specimen setups for the shrinkage test, respectively. The rectangular beam specimens were set on 100-mm-deep soft styrofoam supports whose center-to-center distance was 80 mm, whereas the double-T beam specimen was set on a styrofoam pad on wood supports to prevent bending creep due to self-weight and restraining the shrinkage strain of the test beam.

## 3. Test Results

The four sets of experiments were performed in a laboratory at a constant temperature of 20 °C and a humidity of 60%. All the specimens were covered with non-woven fabric so that they were not exposed to the air and were fully moisturized by sprinkling water until the formworks were removed. The formworks were removed two days after concrete casting for test series A, B, and C, and seven days after casting for test series D. The specimens for A, B, and C were cured in water until one day before the gage attachment work. For series D, the double-T beam specimen was exposed to the laboratory climatic condition to be in the same condition as the two-span double-T beam that was under prestressing work for 30 days. The shrinkage strains for the test series A, B, C, and D were measured from eight days, nine days, seven days, and 35 days after concrete casting, respectively. The tests were ended after 85 days, 63 days, 100 days, and 110 days, for test series A, B, C, and D, respectively. Gage readings from the specimens were automatically stored every minute in a data acquisition system with 81 data channels. All the stored data were extracted from the data acquisition system at five day intervals to avoid accidental data loss. The elastic modulus of the concrete was obtained every two weeks from strain gages attached at the mid-height of cylindrical concrete specimens (150 mm diameter and 300 mm height) at equal angles of 90° to estimate its time-dependent development. The compressive strengths and elastic moduli of the concretes at 28 days for the four test series are listed in Table 3.

**Table 3:** Compressive strengths and elastic moduli of concretes of four test series at 28 days.

Test Series	Compressive Strength (MPa)	Elastic Modulus (MPa)
A	28	25,300
B	28	23,800
C	27.1	24,010
D	35	30,800

### 3.1. Beam Shrinkage Test

#### 3.1.1. Shrinkage Strain of Horizontally-Cast Beam Specimen

**Figure 4:**
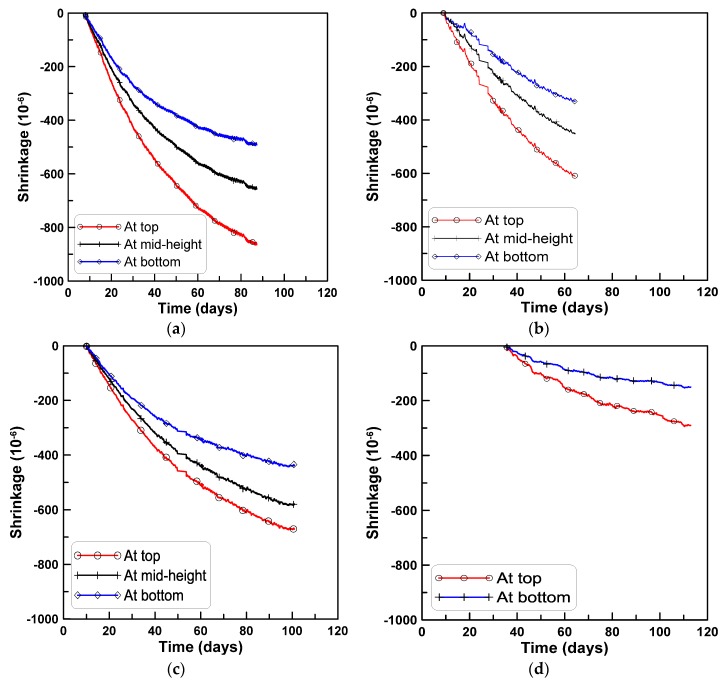
Shrinkage strains of horizontally-cast beam specimens: (**a**) Test series A; (**b**) test series B; (**c**) test series C; (**d**) test series D.

Figure 4a–d shows the average shrinkage strains measured by the surface-attached strain gages at the top, mid-height, and bottom surfaces of the horizontally cast beam specimens of the test series A, B, C, and D, respectively. In the case of test series D, the shrinkage strains were measured at the two locations of the top and bottom surfaces. All four test series show that the measured strains were largest at the top, intermediate at the mid-height, and smallest at the bottom.

**Figure 5:**
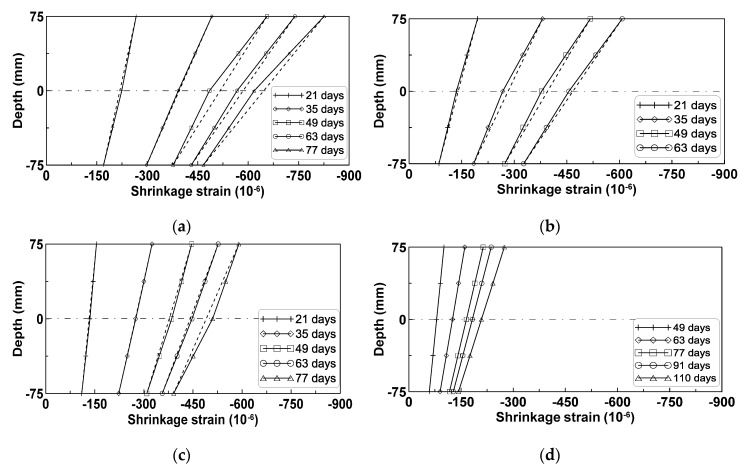
Variation of shrinkage strain distributions within beam depth: (**a**) Test series A; (**b**) test series B; (**c**) test series C; (**d**) test series D.

**Figure 6:**
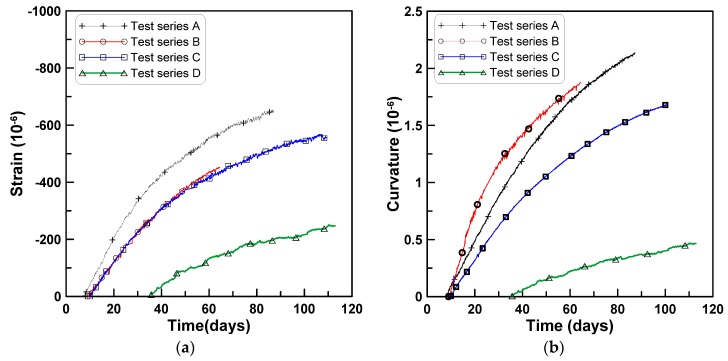
Uniform shrinkage and curvature for four test series: (**a**) Uniform part strain; (**b**) skewed part strain.

Figure 5a–d show the shrinkage strains measured at the three locations at different ages for the four test series A, B, C, and D, respectively. The slopes of the lines connecting the three shrinkage strain measurements show the development of beam curvature due to the variation of shrinkage strain within the beam depth. The variation of shrinkage strain within the beam depth causes a curvature that downwardly deflects the beam. The slopes of the lines connecting the shrinkage strains at the top and bottom in Figure 5 were decomposed into uniform and skewed parts with reference to the centroidal axis. The curvature κ was calculated by dividing the skewed-part strains by the half-height of the test beam where the skewed-part strain was obtained by subtracting the uniform part of the shrinkage strain from the total shrinkage strain measured at the top surface. Figure 6a,b show the uniform parts of the strains and the curvatures for the four test series.

#### 3.1.2. Shrinkage Strain of Horizontally-Cast Beam Specimens

For the vertically cast beam specimens, concrete was placed into the formwork in three stages to prevent the segregation of aggregates, and tamped by a vibrating temper after each placement of fresh concrete, in similar fashion as for the horizontally cast beam specimens. Shrinkage strains were measured from the surface-attached strain gages at the same locations as the horizontally cast test specimens. The measured shrinkage strains were compared to those measured from the horizontally cast test specimens to determine if the variation of shrinkage strains in Figure 4 and Figure 5 were observed in the vertically cast beam specimens.

**Figure 7:**
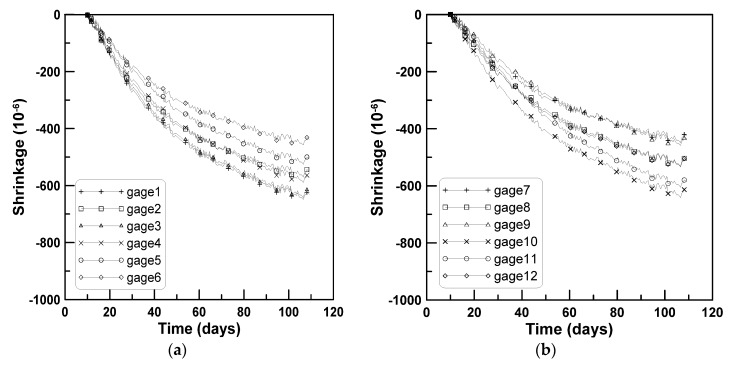
Shrinkage strains of vertically-cast beam specimens of test series C: (**a**) Specimen 1; (**b**) specimen 2.

**Figure 8:**
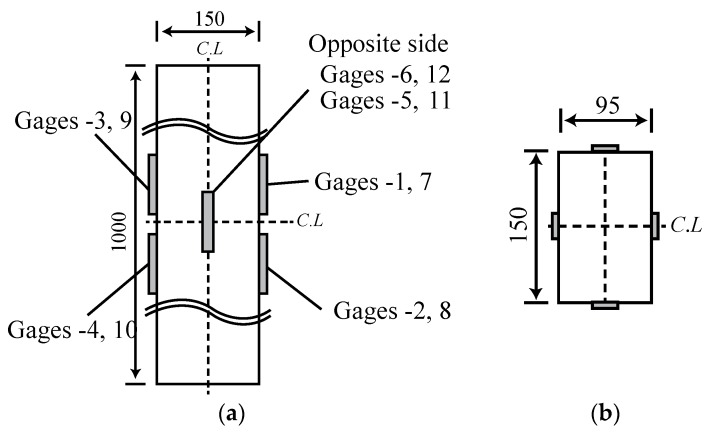
Gage numbering of vertically-cast beam specimens: (**a**) Longitudinal view; (**b**) cross sectional view.

**Figure 9:**
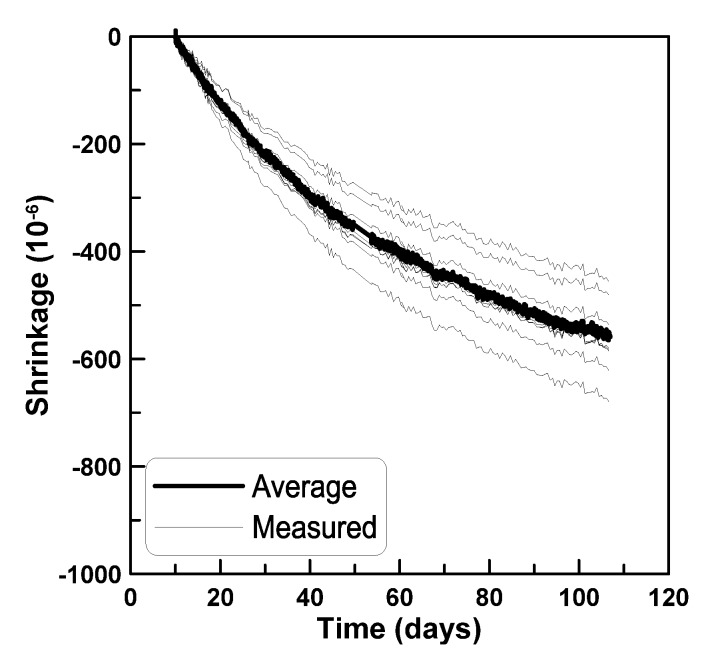
Shrinkage strains measured at the mid-height of cylindrical specimen.

Figure 7 plots the shrinkage strains measured from six surface-attached strain gages (gage numbering is illustrated in Figure 8). In Figure 7, the shrinkage strains measured from the vertically cast specimens range between 0.004 and 0.006 regardless of the locations of the strain gages, in contrast to the observations for horizontally cast beam specimens. Shrinkage strains of cylindrical concrete specimens measuring 150 mm in diameter and 300 mm in height were measured at the mid-height of the specimens to identify the scattering tendency of concrete shrinkage strain. Figure 9 shows that the shrinkage strains measured for the cylindrical concrete specimens range between 0.004 and 0.007, which agrees with the scattering tendency of shrinkage strains measured from the vertically cast beam specimens. The comparison of shrinkage strains measured for horizontally cast and vertically cast beam specimens identifies that the distribution of shrinkage strains within the beam depth occurs in the horizontally cast beam specimens and not in the vertically cast beam specimens.

#### 3.1.3. Shrinkage Strains Measured from Embedded Strain Gages

**Figure 10:**
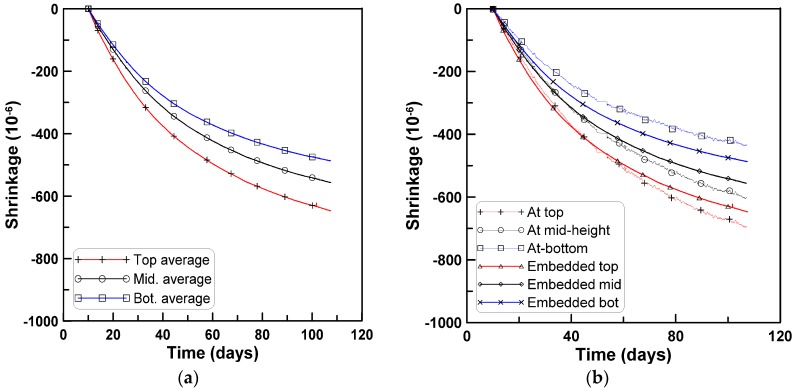
Average shrinkage strains obtained from embedded strain gages (test series C): (**a**) Shrinkage strain measurements; (**b**) comparison with surface-attached gage measurements.

In test series C, embedded strain gages were installed in the interior of beam specimens in addition to the surface-attached strain gages. Figure 10a shows the shrinkage strains measured at the three locations in the interior of the beam specimens shown in Figure 2. The shrinkage strain was largest at the upper, intermediate at the middle, and smallest at the lower gage, which is similar to the findings with surface-attached strain gages. The shrinkage strains measured from the embedded gages are compared to those measured from the surface-attached gages in Figure 10b. The shrinkage strains at the upper and lower embedded gage locations range between the two shrinkage strains at the top and bottom surfaces of the surface-attached gages. The shrinkage strain measured at the mid-height of the surface-attached strain gages is slightly larger than that of the embedded strain gages. This is explained by the fact that shrinkage develops more quickly near the drying surface than in the center of a concrete specimen [8].

### 3.2. Evaluation of Beam Curvature Due to Shrinkage

Shrinkage strains for the four test series in Figure 4 and Figure 5 were decomposed into uniform and skewed parts with reference to the centroidal axis of the test beam specimens to identify the degree of shrinkage strain variation within the beam depth similarly to Figure 6. The skewed part of shrinkage strain is typically neglected in conventional time-dependent analysis of concrete structures. This means that the analysis does not account for the different mix proportions within the beam depth induced during the placement of the fresh concrete.

**Figure 11:**
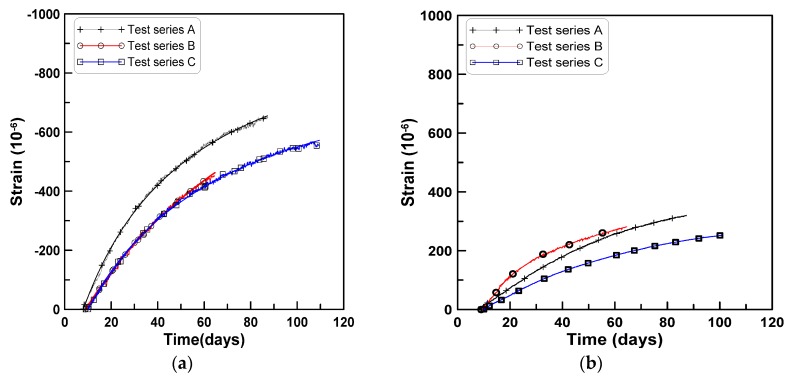
Uniform and skewed parts of shrinkage strains for four test series: (**a**) Uniform part strain; (**b**) skewed part strain.

Figure 11a,b shows the uniform and skewed parts of the shrinkage strains for the test series A, B, and C. Each test data for uniform and skewed parts of shrinkage strains was regressed to obtain the shrinkage parameters defining the fractional function form recommended by ACI 209.2R-08 (2008) as:

(1) $$\varepsilon_{sh}(t,t_{o})=\frac{{\left(t-t_{o}\right)}^{\alpha }}{\beta +{\left(t-t_{o}\right)}^{\alpha }}\varepsilon_{shu}$$

The test results of series D were not included in the regression because the test period and the drying age of series D were different from those of A, B, and C.

The values of coefficients β and α in Equation (1) obtained from the regression analysis are listed in Table 4. The values of $R^{2}$ for coefficient $\beta_{uni}$ were 0.99, 0.992, and 0.998 for test series A, B, and C, respectively, and those for coefficient $\beta_{skew}$ were 0.994, 0.929, and 0.979, for test series A, B, and C, respectively, where $\beta_{uni}$ and $\beta_{uni}$ present the coefficient β for the uniform and skewed parts of shrinkage strain, respectively. In Equation (1), $\varepsilon_{shu}$ is a shrinkage coefficient that defines the amount of strain at an infinite time ($\varepsilon_{shu}=0.78\times 10^{-3}$). For all three cases, the value of $\beta_{uni}$ is less than the value of $\beta_{skew}$. This implies that the uniform part of the shrinkage strain dominates the shrinkage strain development. The ratio of $\beta_{skew}$ to $\beta_{uni}$ is approximately 4.5 for test series A and C, and 1.8 for test series B.

**Table 4:** Values of two coefficients to define uniform and skewed parts of shrinkage strains.

Coefficient		Test Series A	Test Series B	Test Series C
β	$\beta_{uni}$	39.4	73.7	71.3
	$\beta_{skew}$	180.4	133.6	301.2
α		1.13		

## 4. Effect of Aggregate Concentration on Shrinkage Variation

### 4.1. Distribution of Aggregates across the Beam Cross-Section

**Figure 12:**
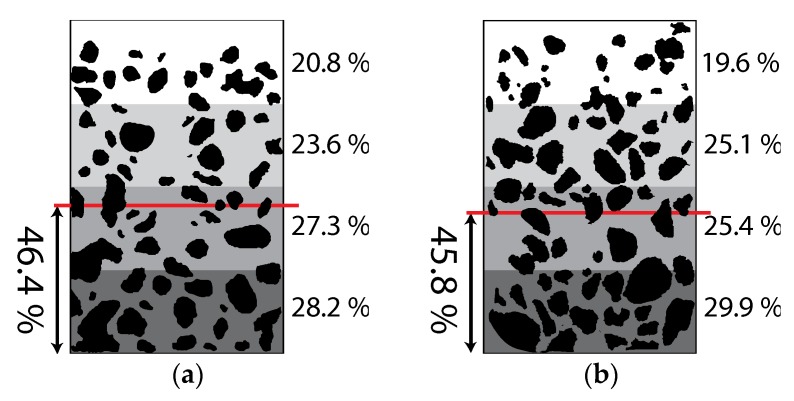
Average percentage of aggregate areas in each sub-area: (**a**) Test series A; (**b**) Test series C.

Test beam specimens of test series A and C were cut into four parts, each 250 mm long, after completion of the test and the distribution of aggregate particles were examined. The cutting plane was divided into four equal sub-areas measuring 37.5 mm in height and 95 mm in width, and the aggregate concentration was measured on each sub-area. Figure 12a,b show the average aggregate concentrations for the four sub-areas of the cutting planes and the locations of the centroids of aggregates for test series A and C, respectively. The calculated aggregate concentrations are listed in Table 5 where $G_{Area}$ represents the location of the centroid of the aggregate areas in the cutting plane and A-1, A-2, A-3, and C-1, C-2, C-3 represent the cutting planes for test series A and C, respectively. The location of centroid $G_{Area}$ was calculated by taking the first moment of each aggregate area on each cutting plane. It is observed from Figure 12 and Table 5 that the aggregate concentration is the lowest at the top sub-area and increases toward the bottom, and the centroid of the aggregate areas on the cutting plane is below the centroid of the test beam, at 46.1% of the beam depth from the bottom surface. This implies that the variation in aggregate concentration within the beam depth came about during placement of the fresh concrete and caused the variation of shrinkage strain within the beam depth.

**Table 5:** Aggregate concentration for each sub-area.

Sub-Area Location	Percentage Ratio (%)							
	Test Specimen in Test Series A				Test Specimen in Test Series C			
	A-1	A-2	A-3	Average	C-1	C-2	C-3	Average
Top	21.5	21.3	18.9	20.8	21.8	19.8	17.4	19.6
Top-mid	26.1	23.2	24.0	23.6	21.1	27	30.3	26.1
Bot-mid	34.6	26.8	24.4	27.3	26.6	21.8	27.8	25.4
Bottom	33.1	28.6	32.8	28.2	30.5	31.4	27.8	29.9
$G_{Area}$	47.8	46.8	44.8	46.4	46	46.3	45.1	45.8

### 4.2. Evaluation of Shrinkage Variation by Hobbs’s Equation

Shrinkage strains within the beam depth of the test specimens were predicted by Hobbs’s equation [10], which takes into account the effects of aggregate volume concentration on shrinkage strain. The uniform parts of the shrinkage strains shown in Figure 11a were used as the reference shrinkage strain for test series A and C. For test series B and D, two sub-areas, above and below the centroid of the test beam were considered, and 5% of the aggregate concentration was subtracted and added, respectively, to the design aggregate concentration of the mix because aggregate concentrations on the cutting plane were not measured for these series.

Hobbs’s equation is expressed as:

(2) $$C_{c}=C_{m}\left[\frac{1-D_{a}}{1+D_{a}}\right]$$

where $C_{c}$, $C_{m}$, and $D_{a}$ are shrinkage strain, shrinkage of the cement paste fraction, and aggregate volume concentration. The shrinkage of the cement paste fraction $C_{m}$ in Equation (2) was calculated by substituting the values of $C_{c}$ and $D_{a}$ into Equation (2), where the uniform part shrinkage strain was used for $C_{c}$ and the aggregate volume fraction of the mix design of Table 1 was used for $D_{a}$. Thus, this calculation approximates the shrinkage character of the test beam using the uniform part of the shrinkage strain. Aggregate volume concentrations $D_{a}$ for the four sub-areas of test series A, B, C, and D were calculated by computing the aggregate volumes corresponding to the aggregate concentration in Table 5 from the total aggregate volumes determined in the mix designs. The values for the aggregate volumes and aggregate volume concentrations for each sub-area are listed in Table 6 where $V_{a}{\text{ (N/m}}^{\text{3}}\text{)}$ is the aggregate volume in a sub-area. For test series B and D, the aggregate volumes $V_{a}$ were not available (see Table 6) because the aggregate concentration was not measured. In these cases, a ±5% fluctuation was imposed on the aggregate volume concentration $D_{a}$.

**Table 6:** Aggregate volume concentration for four test series A, B, C, and D.

Location	Test Series A		Test Series B		Test Series C		Test Series D	
	$V_{a}$	$D_{a}$	$V_{a}$	$D_{a}$	$V_{a}$	$D_{a}$	$V_{a}$	$D_{a}$
Top	6340	0.69	–	0.70	5950	0.68	–	0.69
Top-mid	7190	0.73	–		7620	0.75	–	
Bot-mid	8320	0.78	–	0.80	7710	0.76	–	0.79
Bottom	8590	0.79	–		9070	0.81	–	
Average	–	0.75	–	0.75	–	0.75	–	0.74

**Figure 13:**
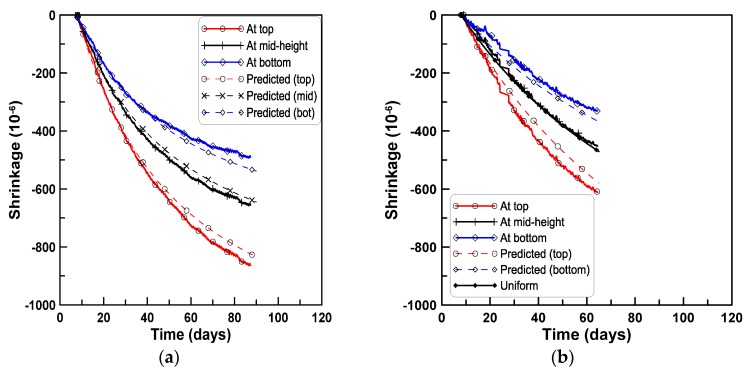

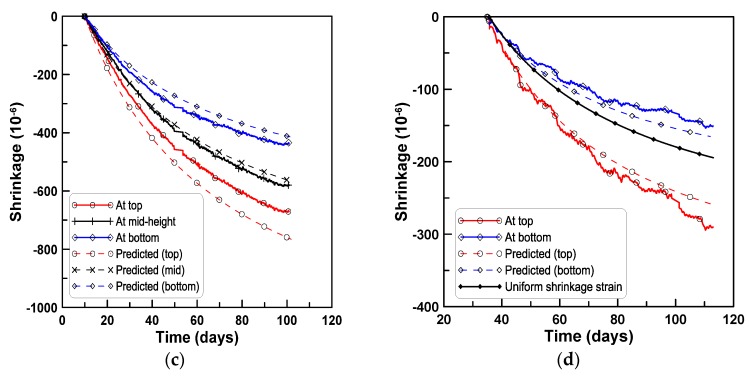
Comparison of measured and predicted shrinkage strains within beam depth for test series A, B, C, and D (test-based): (**a**) Test series A; (**b**) test series B; (**c**) test series C; (**d**) test series D.

Figure 13 compares the measured shrinkage strains at the three strain gage locations, the predicted shrinkage strains based on Hobbs’s equation for the sub-areas within the beam depth, and the uniform shrinkage strains for the four test series. For series A, B, and D, the predicted shrinkage strains at the top and bottom sub-areas are slightly smaller and larger than the measured shrinkage strains at the top and bottom surfaces of test specimens, respectively. In contrast, for series C the predicted shrinkage strains at the top and bottom sub-areas are larger and smaller than the measured shrinkage strains at the top and bottom surfaces of test beam specimens, respectively.

**Figure 14:**
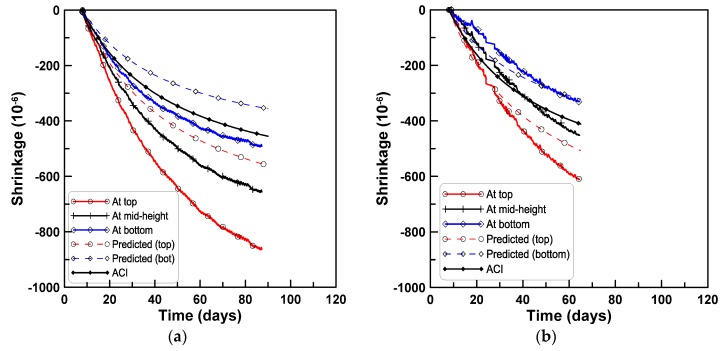

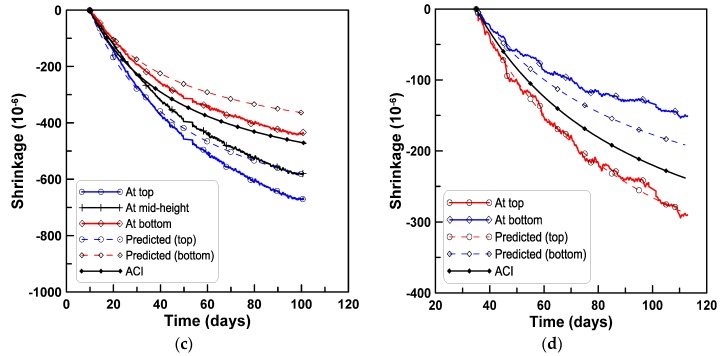
Comparison of measured and predicted shrinkage strains within beam depth for test series A, B, C, and D (ACI-based): (**a**) Test series A; (**b**) test series B; (**c**) test series C; (**d**) test series D.

The shrinkage strains were calculated by the ACI 209 model with consideration of the mix designs and the curing conditions of the test series, to generalize the predicting process for shrinkage strains within the beam depth. Two sub-areas, above and below the centroid of the test beam, were considered, and 5% of aggregate volume concentration was subtracted and added, respectively, to the design aggregate volume concentration of the mix. The shrinkage strains accounting for the change of aggregate concentration within the beam depth were calculated by Hobbs’s equation. Figure 14a–d compares the measured shrinkage strains at the top and bottom surfaces of test specimens with the predicted shrinkage strains at the two sub-areas for the four test series of A, B, C, and D, respectively. The mismatch between the measured and predicted shrinkage strains is primarily due to the difference between the measured uniform shrinkage strains and the shrinkage strains by the ACI model. Figure 13 and Figure 14 demonstrate that the variation of shrinkage strain within the beam depth is caused by the variation of the aggregate volume concentration within the beam depth, and the variation of shrinkage strain within the beam depth can be estimated by accounting for the change of aggregate volume concentration in Hobbs’s equation.

## 5. Conclusions

The variation of shrinkage strain within the beam depth dimension was examined through four series of time-dependent laboratory experiments on unreinforced concrete beam specimens. Two types of beam specimens, horizontally cast and vertically cast, were tested to examine if the aggregate concentration changes during placing the fresh concrete. Two types of strain gages, surface-attached and embedded strain gages, were used to measure shrinkage strains within the depth dimension of the test beam. The test beams were cut into four sections after completion of the test, and the cutting planes were divided into four equal sub-areas to measure the aggregate concentrations for each sub-area. The variation of shrinkage strain within beam depth was investigated by applying the measured aggregate concentrations to Hobbs’s equation. Based on the results of the study, the following conclusions were made:

1. Shrinkage strains measured from the horizontally cast beam specimens varied within the beam depth, increasing toward the top of the beam. Comparison of shrinkage strains measured for the vertically cast beam specimens and cylindrical specimens showed a similar scatter, which can be explained by the probabilistic uncertainty of concrete properties.
2. Shrinkage strains measured from the embedded gages showed a similar tendency to those measured from the surface-attached gages with regard to the distribution of shrinkage strain within the beam depth.
3. The measured shrinkage strain was decomposed into uniform and skewed parts with reference to the centroid of the test beam specimen. The skewed part of the shrinkage strain was sufficiently large compared to the magnitude of the uniform part of the shrinkage strain.
4. The aggregate concentration was measured for four equal sub-areas in the cutting plane of the test beam specimen. The percentage ratios of aggregate concentration for each sub-area identified the variation of aggregate concentration within the beam depth, and the aggregate concentration increased toward the bottom of the beam depth.
5. The shrinkage strains of the test beam specimens within the beam depth were predicted by Hobbs’s equation by taking the variation of aggregate concentration into account. Comparison of the measured shrinkage strain distribution with the shrinkage strain predicted by applying aggregate volume concentration showed a reasonable agreement. This presents a way of computing the skewed part of shrinkage strain with the code-specified shrinkage model, as well as providing information about the mix design.
